# Crystal structures of salen-type ligands 2-[(1*E*)-({1-(3-chloro­phen­yl)-2-[(*E*)-(2-hy­droxy­benzyl­idene)amino]­prop­yl}imino)­meth­yl]phenol and 2-[(1*E*)-({1-(4-chloro­phen­yl)-2-[(*E*)-(2-hy­droxy­benzyl­idene)amino]­prop­yl}imino)­meth­yl]phenol

**DOI:** 10.1107/S2056989017016292

**Published:** 2017-11-17

**Authors:** A. Gayathri, K. Rajeswari, T. Vidhyasagar, S. Selvanayagam

**Affiliations:** a Department of Chemistry, Annamalai University, Annamalainagar, Chidambaram 608 002, India; b PG & Research Department of Physics, Government Arts College, Melur 625 106, India

**Keywords:** crystal structure, salen ligand, schiff base, O—H⋯N intra­molecular hydrogen bonds, C—H⋯O and C—H⋯π inter­molecular inter­actions

## Abstract

The title compounds, C_23_H_21_ClN_2_O_2_, differ from each other only by the position of the Cl atom on the corresponding benzene ring: *meta* relative to the central *sp* ^3^ C atom for (I) and *para* for (II). In (I), the hy­droxy­phenyl rings are almost parallel, the dihedral angle between the mean planes being 9.2 (2)°, but in (II), the relative position of the ring is different, characterized by a dihedral angle of 48.5 (1)°. Compound (I) features intra­molecular O—H⋯N and inter­molecular C—H⋯O hydrogen bonds, while in (II), intra­molecular O—H⋯N, C—H⋯N hydrogen bonds and weak inter­molecular C—H⋯π inter­actions are observed. Compound (I) was refined as an inversion twin.

## Abstract 2

The title compounds are differing only by the position of the chlorine atom in the benzene ring. The mol­ecular structures are very similar, except for the relative position of the hy­droxy­phenyl rings.

## Chemical context   

Salen-type Schiff bases possessing an unsymmetrical vicinal di­amine backbone are promising candidates in synthetic and material science research. Salen ligands and their complexes are widely studied for their extensive applications in various fields, for their luminescent property (Chakraborty *et al.*, 2015 ▸; Chen *et al.*, 2013 ▸), photophysical properties (Cheng *et al.*, 2013 ▸), NLO activity (Nayar & Ravikumar, 2014 ▸; Zeyrek, 2013 ▸) *etc*. Recent reports on a single-crystal study (Habibi *et al.*, 2007 ▸), spectroscopic and DFT calculations (de Toledo *et al.*, 2015 ▸) and the utility in asymmetric syntheses (Yang *et al.*, 2011 ▸) of this type of ligand address the novelty of these compounds and speak of the impact of their efficacy. In view of the importance of the title compounds, we have undertaken a single-crystal X-ray diffraction study and the results are presented here.
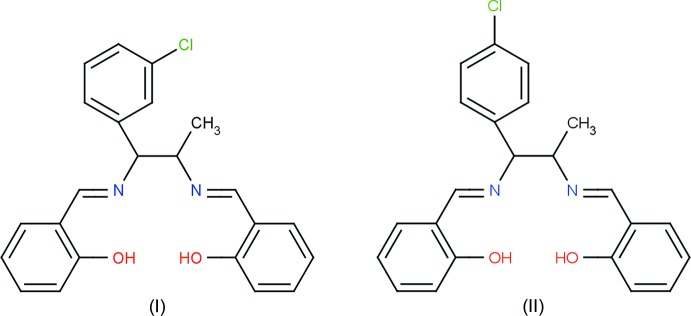


## Structural commentary   

**Figure 1:**
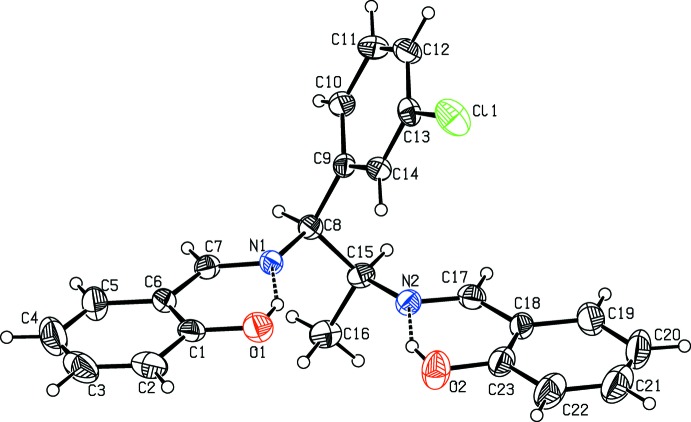
A view of the mol­ecular structure of compound (I), showing the atom labelling. Displacement ellipsoids are drawn at the 30% probability level. Dashed lines represent intra­molecular O—H⋯N hydrogen bonds (Table 1 ▸).

**Figure 3:**
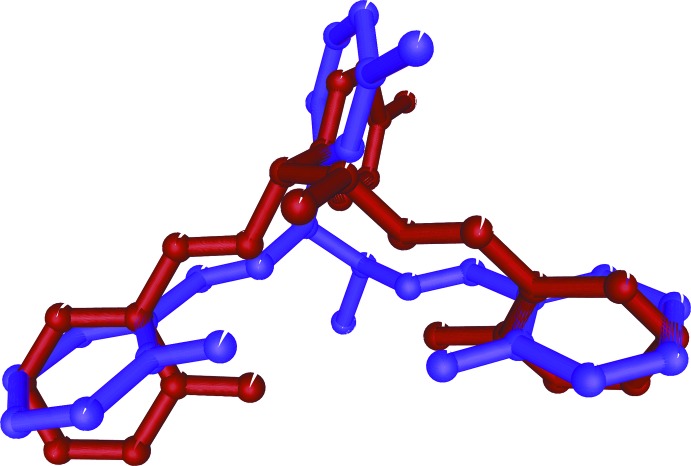
Superposition (excluding atom Cl1) of compound (I) (blue) and compound (II) (red).

The mol­ecular structure of the title compounds, (I) and (II), are illustrated in Figs. 1 ▸ and 2 ▸, respectively. Fig. 3 ▸ shows a superposition of the two compounds except for Cl1 using *Qmol* (Gans & Shalloway, 2001 ▸); the r.m.s. deviation is 2.3 Å. Compound (I) has two chiral centers with the absolute configuration determined as C8(*S*), C15(*S*). The chloro­phenyl group is almost planar with atom Cl1 deviating by 0.013 (1) Å from the ring in (I) whereas in (II) the chlorine atom deviates by 0.079 (1) Å. In (I), hy­droxy atoms O1 and O2 deviate by 0.051 (3) and 0.012 (3) Å, respectively, from the phenyl ring to which they are attached. In (II), hy­droxy atoms O1 and O2 deviate by 0.006 (2) and 0.002 (2) Å, respectively, from the ring. The dihedral angle between these two rings is 9.2 (2)° in (I) and 48.5 (1)° in (II).

In compounds (I) and (II), the mol­ecular structure maybe influenced by two intra­molecular O—H⋯N hydrogen bonds (Tables 1 ▸ and 2 ▸). These two hydrogen bonds form 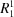
(6) ring motifs; see Figs. 1 ▸ and 2 ▸. C—H⋯N intra­molecular hydrogen bonds are also observed in compound (II).

## Supra­molecular features   

**Figure 4:**
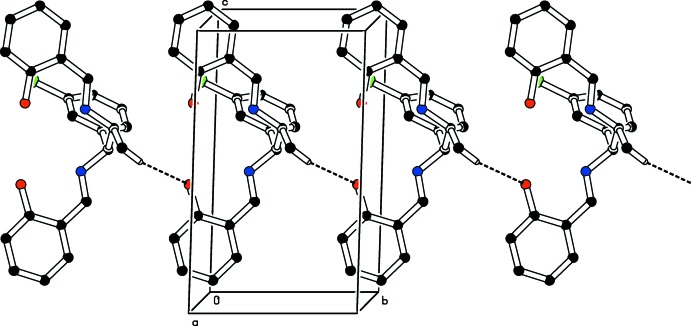
The crystal packing of the title compound (I) viewed along the *a* axis. The C—H⋯O hydrogen bonds are shown as dashed lines (see Table 1 ▸). For clarity, H atoms not involved in these inter­actions have been omitted.

**Figure 5:**
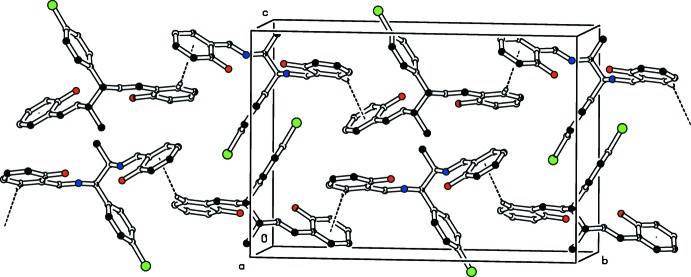
The crystal packing of the title compound (II) viewed along the *a* axis. The C—H⋯π inter­actions are shown as dashed lines. For clarity, H atoms not involved in these inter­actions have been omitted.

In the crystal of (I), C—H⋯O inter­actions link the mol­ecules to form *C*(9) chains propagating along [010]; see Fig. 4 ▸ and Table 1 ▸. In compound (II), the mol­ecules are connected only by C—H⋯π inter­actions, which form *C*(11) chains propagating along the *ab* plane of the unit cell; see Fig. 5 ▸.

## Synthesis and crystallization   

The synthesis of the salen ligand 2-[(1*E*)-({1-(3-chloro­phen­yl)-2-[(*E*)-(2-hy­droxy­benzyl­idene)amino]­prop­yl}imino)­meth­yl]phenol was achieved by the condensation of salicyl­aldehyde (0.02 mol) and 1-(3-chloro­phen­yl)propane-1,2-di­amine (0.01 mol) in ethanol (25 ml, 99%). The completion of the reaction was monitored by TLC. The obtained yellow solid was purified by recrystallization from ethanol. Single crystals suitable for X-ray analysis were obtained by slow evaporation from ethanol. The above procedure was repeated with 1-(4-chloro­phen­yl)propane-1,2-di­amine (0.01 mol) instead of 1-(3-chloro­phen­yl)propane-1,2-di­amine to synthesise 2-[(1*E*)-({1-(4-chloro­phen­yl)-2-[(*E*)-(2-hy­droxy­benzyl­idene)amino]­prop­yl}imino)­meth­yl]phenol.

## Refinement   

Crystal data, data collection and structure refinement details are summarized in Table 3 ▸. In both compounds, hy­droxy H atoms H1 and H2 were located from difference-Fourier maps. All other H atoms were placed in idealized positions and allowed to ride on their parent atoms: C—H = 0.93–0.97 Å, with *U*_iso_(H) = 1.5*U*_eq_(C) for methyl H atoms and 1.2*U*_eq_(C) for other H atoms. Pairs of O—H bond distances were restrained to 0.82 (1) Å. Compound (I) was refined as an inversion twin.

## Supplementary Material

Crystal structure: contains datablock(s) I, II, global. DOI: 10.1107/S2056989017016292/zq2239sup1.cif

Structure factors: contains datablock(s) I. DOI: 10.1107/S2056989017016292/zq2239Isup2.hkl

Structure factors: contains datablock(s) II. DOI: 10.1107/S2056989017016292/zq2239IIsup3.hkl

CCDC references: 1412946, 1412945

Additional supporting information:  crystallographic information; 3D view; checkCIF report

## Figures and Tables

**Figure 2:**
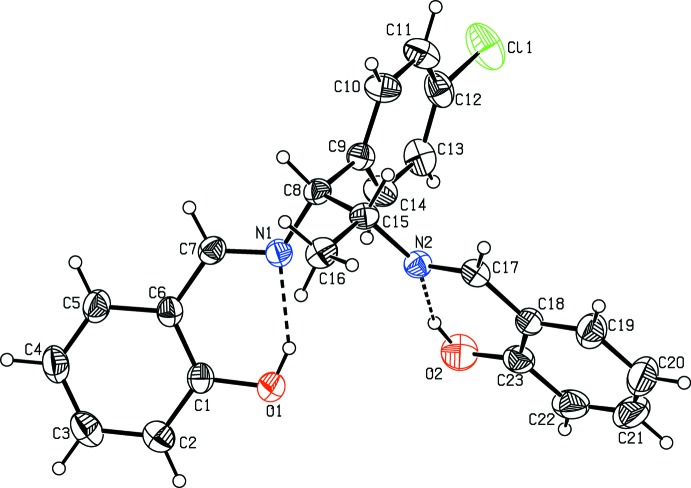
A view of the mol­ecular structure of compound (II), showing the atom labelling. Displacement ellipsoids are drawn at the 30% probability level. Dashed lines represent intra­molecular O—H⋯N hydrogen bonds (Table 2 ▸).

**Table 1:** Hydrogen-bond geometry (Å, °) for (I)

*D*—H⋯*A*	*D*—H	H⋯*A*	*D*⋯*A*	*D*—H⋯*A*
O1—H1⋯N1	0.82 (1)	1.88 (3)	2.596 (3)	146 (4)
O2—H2⋯N2	0.82 (1)	1.88 (3)	2.588 (3)	143 (4)
C16—H16*C*⋯O1^i^	0.96	2.52	3.448 (4)	161

Symmetry code: (i) 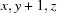.

**Table 2:** Hydrogen-bond geometry (Å, °) for (II) *Cg* is the centroid of the C18–C23 ring.

*D*—H⋯*A*	*D*—H	H⋯*A*	*D*⋯*A*	*D*—H⋯*A*
O1—H1⋯N1	0.83 (1)	1.83 (2)	2.589 (2)	150 (3)
O2—H2⋯N2	0.82 (1)	1.81 (2)	2.557 (3)	150 (3)
C14—H14⋯N1	0.93	2.52	2.845 (3)	101
C5—H5⋯*Cg* ^i^	0.93	2.76	3.449 (3)	132

Symmetry code: (i) 
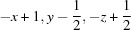
.

**Table 3:** Experimental details

	(I)	(II)
Crystal data		
Chemical formula	C_23_H_21_ClN_2_O_2_	C_23_H_21_ClN_2_O_2_
*M*_r_	392.87	392.87
Crystal system, space group	Monoclinic, *P*2_1_	Monoclinic, *P*2_1_/*c*
Temperature (K)	296	296
*a*, *b*, *c* (Å)	12.8126 (7), 7.0224 (3), 12.8169 (6)	6.7923 (2), 20.8261 (8), 14.1744 (6)
β (°)	117.207 (3)	92.435 (2)
*V* (Å^3^)	1025.61 (9)	2003.26 (13)
*Z*	2	4
Radiation type	Mo *K*α	Mo *K*α
μ (mm^−1^)	0.21	0.21
Crystal size (mm)	0.35 × 0.30 × 0.25	0.35 × 0.30 × 0.25

Data collection		
Diffractometer	Bruker Kappa APEXII CCD	Bruker Kappa APEXII CCD
Absorption correction	Multi-scan (*SADABS*; Bruker, 2004 ▸)	Multi-scan (*SADABS*; Bruker, 2004 ▸)
*T*_min_, *T*_max_	0.921, 0.959	0.927, 0.959
No. of measured, independent and observed [*I* > 2σ(*I*)] reflections	15137, 4892, 2738	42044, 4182, 2599
*R*_int_	0.039	0.044
(sin θ/λ)_max_ (Å^−1^)	0.679	0.629

Refinement		
*R*[*F* ^2^ > 2σ(*F* ^2^)], *wR*(*F* ^2^), *S*	0.044, 0.087, 0.98	0.047, 0.140, 1.08
No. of reflections	4892	4182
No. of parameters	263	262
No. of restraints	3	2
H-atom treatment	H atoms treated by a mixture of independent and constrained refinement	H atoms treated by a mixture of independent and constrained refinement
Δρ_max_, Δρ_min_ (e Å^−3^)	0.16, −0.21	0.30, −0.29
Absolute structure	Refined as an inversion twin	–
Absolute structure parameter	0.27 (8)	–

Computer programs: *APEX2*, *SAINT* and *XPREP* (Bruker, 2004 ▸), *SHELXS97* (Sheldrick, 2008 ▸), *SHELXL2014* (Sheldrick, 2015 ▸), *ORTEP-3 for Windows* (Farrugia, 2012 ▸) and *PLATON* (Spek, 2009 ▸).

## supplementary crystallographic information

### 2-[(1E)-({1-(3-Chlorophenyl)-2-[(E)-(2-hydroxybenzylidene)amino]propyl}imino)methyl]phenol (I) Crystal data

**Table:**

C_23_H_21_ClN_2_O_2_	*F*(000) = 412
*M**_r_* = 392.87	*D*_x_ = 1.272 Mg m^−^^3^
Monoclinic, *P*2_1_	Mo *K*α radiation, λ = 0.71073 Å
*a* = 12.8126 (7) Å	Cell parameters from 4564 reflections
*b* = 7.0224 (3) Å	θ = 3.1–28.7°
*c* = 12.8169 (6) Å	µ = 0.21 mm^−^^1^
β = 117.207 (3)°	*T* = 296 K
*V* = 1025.61 (9) Å^3^	Block, yellow
*Z* = 2	0.35 × 0.30 × 0.25 mm

### 2-[(1E)-({1-(3-Chlorophenyl)-2-[(E)-(2-hydroxybenzylidene)amino]propyl}imino)methyl]phenol (I) Data collection

**Table:**

Bruker Kappa APEXII CCD diffractometer	4892 independent reflections
Radiation source: fine-focus sealed tube	2738 reflections with *I* > 2σ(*I*)
Graphite monochromator	*R*_int_ = 0.039
ω and φ scan	θ_max_ = 28.9°, θ_min_ = 3.1°
Absorption correction: multi-scan (SADABS; Bruker, 2004)	*h* = −17→17
*T*_min_ = 0.921, *T*_max_ = 0.959	*k* = −9→9
15137 measured reflections	*l* = −17→17

### 2-[(1E)-({1-(3-Chlorophenyl)-2-[(E)-(2-hydroxybenzylidene)amino]propyl}imino)methyl]phenol (I) Refinement

**Table:**

Refinement on *F*^2^	Hydrogen site location: mixed
Least-squares matrix: full	H atoms treated by a mixture of independent and constrained refinement
*R*[*F*^2^ > 2σ(*F*^2^)] = 0.044	*w* = 1/[σ^2^(*F*_o_^2^) + (0.0355*P*)^2^] where *P* = (*F*_o_^2^ + 2*F*_c_^2^)/3
*wR*(*F*^2^) = 0.087	(Δ/σ)_max_ < 0.001
*S* = 0.98	Δρ_max_ = 0.16 e Å^−^^3^
4892 reflections	Δρ_min_ = −0.21 e Å^−^^3^
263 parameters	Absolute structure: Refined as an inversion twin
3 restraints	Absolute structure parameter: 0.27 (8)

### 2-[(1E)-({1-(3-Chlorophenyl)-2-[(E)-(2-hydroxybenzylidene)amino]propyl}imino)methyl]phenol (I) Special details

**Table:**

Geometry. All esds (except the esd in the dihedral angle between two l.s. planes) are estimated using the full covariance matrix. The cell esds are taken into account individually in the estimation of esds in distances, angles and torsion angles; correlations between esds in cell parameters are only used when they are defined by crystal symmetry. An approximate (isotropic) treatment of cell esds is used for estimating esds involving l.s. planes.
Refinement. Refined as a 2-component inversion twin.

### 2-[(1E)-({1-(3-Chlorophenyl)-2-[(E)-(2-hydroxybenzylidene)amino]propyl}imino)methyl]phenol (I) Fractional atomic coordinates and isotropic or equivalent isotropic displacement parameters (Å^2^)

**Table:**

	*x*	*y*	*z*	*U*_iso_*/*U*_eq_	
Cl1	0.12595 (9)	−0.08259 (13)	0.75791 (8)	0.0764 (3)	
O1	0.2698 (2)	−0.1099 (3)	0.4005 (2)	0.0608 (6)	
H1	0.267 (4)	−0.021 (4)	0.440 (3)	0.093 (15)*	
O2	0.5035 (2)	−0.0707 (4)	0.7066 (2)	0.0756 (7)	
H2	0.460 (3)	0.018 (4)	0.671 (3)	0.094 (15)*	
N1	0.2403 (2)	0.2414 (3)	0.4445 (2)	0.0392 (6)	
N2	0.4325 (2)	0.2792 (3)	0.6800 (2)	0.0447 (6)	
C1	0.2232 (3)	−0.0598 (5)	0.2872 (3)	0.0484 (8)	
C2	0.2161 (3)	−0.1952 (6)	0.2053 (4)	0.0696 (11)	
H2A	0.2458	−0.3170	0.2295	0.084*	
C3	0.1651 (4)	−0.1487 (7)	0.0886 (4)	0.0826 (13)	
H3	0.1599	−0.2402	0.0340	0.099*	
C4	0.1219 (4)	0.0292 (8)	0.0510 (4)	0.0901 (14)	
H4	0.0859	0.0578	−0.0286	0.108*	
C5	0.1319 (3)	0.1664 (6)	0.1320 (3)	0.0696 (10)	
H5	0.1040	0.2887	0.1065	0.083*	
C6	0.1828 (3)	0.1248 (4)	0.2505 (3)	0.0457 (8)	
C7	0.1946 (2)	0.2720 (4)	0.3347 (3)	0.0425 (7)	
H7	0.1676	0.3939	0.3071	0.051*	
C8	0.2472 (2)	0.3981 (4)	0.5223 (2)	0.0400 (7)	
H8	0.2130	0.5111	0.4737	0.048*	
C9	0.1745 (2)	0.3515 (4)	0.5843 (2)	0.0376 (7)	
C10	0.1000 (3)	0.4861 (4)	0.5917 (3)	0.0472 (8)	
H10	0.0941	0.6051	0.5576	0.057*	
C11	0.0340 (3)	0.4461 (5)	0.6495 (3)	0.0586 (9)	
H11	−0.0158	0.5386	0.6537	0.070*	
C12	0.0411 (3)	0.2721 (5)	0.7004 (3)	0.0539 (8)	
H12	−0.0032	0.2453	0.7393	0.065*	
C13	0.1150 (3)	0.1387 (4)	0.6927 (2)	0.0444 (8)	
C14	0.1816 (2)	0.1744 (4)	0.6362 (2)	0.0413 (7)	
H14	0.2312	0.0809	0.6325	0.050*	
C15	0.3754 (2)	0.4435 (4)	0.6076 (3)	0.0437 (7)	
H15	0.3765	0.5478	0.6589	0.052*	
C16	0.4443 (3)	0.5057 (4)	0.5432 (3)	0.0568 (9)	
H16A	0.4503	0.4010	0.4981	0.085*	
H16B	0.5215	0.5454	0.5991	0.085*	
H16C	0.4045	0.6098	0.4918	0.085*	
C17	0.4838 (3)	0.2952 (4)	0.7907 (3)	0.0495 (8)	
H17	0.4803	0.4115	0.8237	0.059*	
C18	0.5477 (3)	0.1402 (5)	0.8680 (3)	0.0492 (8)	
C19	0.6041 (3)	0.1668 (6)	0.9894 (3)	0.0740 (11)	
H19	0.5978	0.2828	1.0211	0.089*	
C20	0.6697 (4)	0.0201 (8)	1.0629 (4)	0.0911 (14)	
H20	0.7092	0.0385	1.1437	0.109*	
C21	0.6755 (4)	−0.1514 (8)	1.0155 (5)	0.0927 (14)	
H21	0.7182	−0.2500	1.0652	0.111*	
C22	0.6206 (3)	−0.1822 (6)	0.8973 (4)	0.0805 (12)	
H22	0.6265	−0.2998	0.8671	0.097*	
C23	0.5557 (3)	−0.0364 (5)	0.8225 (3)	0.0567 (9)	

### 2-[(1E)-({1-(3-Chlorophenyl)-2-[(E)-(2-hydroxybenzylidene)amino]propyl}imino)methyl]phenol (I) Atomic displacement parameters (Å^2^)

**Table:**

	*U*^11^	*U*^22^	*U*^33^	*U*^12^	*U*^13^	*U*^23^
Cl1	0.1129 (8)	0.0564 (5)	0.0816 (7)	−0.0087 (6)	0.0632 (6)	0.0050 (5)
O1	0.0708 (16)	0.0535 (15)	0.0610 (17)	0.0124 (12)	0.0325 (14)	0.0025 (13)
O2	0.096 (2)	0.0583 (16)	0.0555 (17)	0.0182 (16)	0.0201 (15)	0.0044 (15)
N1	0.0428 (15)	0.0390 (14)	0.0373 (16)	0.0061 (11)	0.0197 (13)	0.0049 (11)
N2	0.0425 (15)	0.0451 (15)	0.0438 (18)	0.0026 (12)	0.0173 (14)	0.0009 (13)
C1	0.0370 (18)	0.064 (2)	0.050 (2)	−0.0029 (17)	0.0250 (17)	−0.0075 (19)
C2	0.062 (2)	0.074 (2)	0.086 (3)	−0.009 (2)	0.046 (2)	−0.021 (2)
C3	0.078 (3)	0.109 (4)	0.075 (3)	−0.029 (3)	0.048 (3)	−0.045 (3)
C4	0.092 (3)	0.130 (4)	0.047 (3)	−0.020 (3)	0.030 (3)	−0.019 (3)
C5	0.071 (3)	0.091 (3)	0.044 (2)	−0.001 (2)	0.023 (2)	0.004 (2)
C6	0.0387 (19)	0.063 (2)	0.038 (2)	−0.0010 (16)	0.0197 (16)	0.0002 (17)
C7	0.0394 (18)	0.0428 (17)	0.047 (2)	0.0040 (15)	0.0214 (16)	0.0106 (16)
C8	0.0457 (18)	0.0336 (15)	0.0426 (17)	0.0089 (14)	0.0220 (15)	0.0074 (15)
C9	0.0373 (17)	0.0402 (17)	0.0320 (16)	0.0044 (13)	0.0129 (15)	−0.0016 (13)
C10	0.049 (2)	0.0468 (19)	0.047 (2)	0.0117 (14)	0.0228 (17)	0.0036 (14)
C11	0.053 (2)	0.065 (2)	0.064 (2)	0.0155 (17)	0.0332 (18)	−0.0042 (19)
C12	0.051 (2)	0.067 (2)	0.053 (2)	−0.0044 (19)	0.0318 (18)	−0.0093 (18)
C13	0.051 (2)	0.0461 (17)	0.0346 (18)	−0.0079 (16)	0.0185 (16)	−0.0057 (15)
C14	0.0426 (19)	0.0418 (17)	0.0412 (18)	0.0036 (14)	0.0206 (16)	−0.0035 (14)
C15	0.0427 (19)	0.0355 (17)	0.0521 (19)	0.0031 (15)	0.0211 (16)	−0.0009 (15)
C16	0.055 (2)	0.0485 (18)	0.072 (3)	0.0040 (16)	0.034 (2)	0.0124 (17)
C17	0.0440 (19)	0.056 (2)	0.052 (2)	−0.0053 (16)	0.0257 (18)	−0.0095 (18)
C18	0.0395 (19)	0.068 (2)	0.038 (2)	−0.0039 (17)	0.0165 (17)	0.0065 (18)
C19	0.062 (3)	0.108 (3)	0.050 (2)	−0.015 (2)	0.025 (2)	−0.002 (2)
C20	0.068 (3)	0.142 (4)	0.046 (3)	−0.010 (3)	0.011 (2)	0.027 (3)
C21	0.064 (3)	0.115 (4)	0.079 (4)	0.002 (3)	0.016 (3)	0.040 (3)
C22	0.067 (3)	0.082 (3)	0.077 (3)	0.010 (2)	0.020 (2)	0.031 (2)
C23	0.047 (2)	0.066 (3)	0.051 (2)	−0.0002 (17)	0.0165 (18)	0.0131 (19)

### 2-[(1E)-({1-(3-Chlorophenyl)-2-[(E)-(2-hydroxybenzylidene)amino]propyl}imino)methyl]phenol (I) Geometric parameters (Å, º)

**Table:**

Cl1—C13	1.740 (3)	C10—C11	1.384 (4)
O1—C1	1.341 (3)	C10—H10	0.9300
O1—H1	0.820 (13)	C11—C12	1.369 (4)
O2—C23	1.343 (4)	C11—H11	0.9300
O2—H2	0.823 (13)	C12—C13	1.368 (4)
N1—C7	1.271 (3)	C12—H12	0.9300
N1—C8	1.461 (3)	C13—C14	1.371 (4)
N2—C17	1.266 (4)	C14—H14	0.9300
N2—C15	1.452 (4)	C15—C16	1.522 (4)
C1—C2	1.388 (4)	C15—H15	0.9800
C1—C6	1.395 (4)	C16—H16A	0.9600
C2—C3	1.370 (6)	C16—H16B	0.9600
C2—H2A	0.9300	C16—H16C	0.9600
C3—C4	1.362 (6)	C17—C18	1.448 (4)
C3—H3	0.9300	C17—H17	0.9300
C4—C5	1.379 (5)	C18—C23	1.394 (4)
C4—H4	0.9300	C18—C19	1.396 (4)
C5—C6	1.383 (4)	C19—C20	1.389 (6)
C5—H5	0.9300	C19—H19	0.9300
C6—C7	1.452 (4)	C20—C21	1.367 (6)
C7—H7	0.9300	C20—H20	0.9300
C8—C9	1.510 (4)	C21—C22	1.364 (6)
C8—C15	1.534 (4)	C21—H21	0.9300
C8—H8	0.9800	C22—C23	1.389 (4)
C9—C10	1.377 (4)	C22—H22	0.9300
C9—C14	1.394 (4)		
			
C1—O1—H1	111 (3)	C13—C12—H12	120.9
C23—O2—H2	110 (3)	C11—C12—H12	120.9
C7—N1—C8	118.8 (2)	C12—C13—C14	122.1 (3)
C17—N2—C15	119.8 (2)	C12—C13—Cl1	118.9 (2)
O1—C1—C2	118.8 (3)	C14—C13—Cl1	119.0 (2)
O1—C1—C6	121.5 (3)	C13—C14—C9	119.7 (3)
C2—C1—C6	119.7 (3)	C13—C14—H14	120.2
C3—C2—C1	119.7 (4)	C9—C14—H14	120.2
C3—C2—H2A	120.1	N2—C15—C16	109.1 (2)
C1—C2—H2A	120.1	N2—C15—C8	110.7 (2)
C4—C3—C2	121.3 (4)	C16—C15—C8	111.8 (2)
C4—C3—H3	119.4	N2—C15—H15	108.4
C2—C3—H3	119.4	C16—C15—H15	108.4
C3—C4—C5	119.5 (4)	C8—C15—H15	108.4
C3—C4—H4	120.3	C15—C16—H16A	109.5
C5—C4—H4	120.3	C15—C16—H16B	109.5
C4—C5—C6	120.9 (4)	H16A—C16—H16B	109.5
C4—C5—H5	119.5	C15—C16—H16C	109.5
C6—C5—H5	119.5	H16A—C16—H16C	109.5
C5—C6—C1	118.9 (3)	H16B—C16—H16C	109.5
C5—C6—C7	120.2 (3)	N2—C17—C18	122.8 (3)
C1—C6—C7	121.0 (3)	N2—C17—H17	118.6
N1—C7—C6	122.8 (3)	C18—C17—H17	118.6
N1—C7—H7	118.6	C23—C18—C19	119.0 (3)
C6—C7—H7	118.6	C23—C18—C17	120.6 (3)
N1—C8—C9	109.7 (2)	C19—C18—C17	120.3 (3)
N1—C8—C15	110.7 (2)	C20—C19—C18	120.1 (4)
C9—C8—C15	112.8 (2)	C20—C19—H19	120.0
N1—C8—H8	107.8	C18—C19—H19	120.0
C9—C8—H8	107.8	C21—C20—C19	119.4 (4)
C15—C8—H8	107.8	C21—C20—H20	120.3
C10—C9—C14	118.4 (3)	C19—C20—H20	120.3
C10—C9—C8	120.1 (2)	C22—C21—C20	121.9 (4)
C14—C9—C8	121.6 (2)	C22—C21—H21	119.1
C9—C10—C11	120.7 (3)	C20—C21—H21	119.1
C9—C10—H10	119.7	C21—C22—C23	119.4 (4)
C11—C10—H10	119.7	C21—C22—H22	120.3
C12—C11—C10	120.9 (3)	C23—C22—H22	120.3
C12—C11—H11	119.6	O2—C23—C22	118.0 (3)
C10—C11—H11	119.6	O2—C23—C18	121.8 (3)
C13—C12—C11	118.3 (3)	C22—C23—C18	120.1 (4)
			
O1—C1—C2—C3	177.8 (3)	C11—C12—C13—Cl1	179.5 (2)
C6—C1—C2—C3	−2.6 (5)	C12—C13—C14—C9	−0.1 (4)
C1—C2—C3—C4	0.6 (6)	Cl1—C13—C14—C9	−179.5 (2)
C2—C3—C4—C5	1.5 (7)	C10—C9—C14—C13	0.0 (4)
C3—C4—C5—C6	−1.5 (6)	C8—C9—C14—C13	179.3 (3)
C4—C5—C6—C1	−0.6 (5)	C17—N2—C15—C16	110.4 (3)
C4—C5—C6—C7	178.8 (3)	C17—N2—C15—C8	−126.2 (3)
O1—C1—C6—C5	−177.8 (3)	N1—C8—C15—N2	−60.4 (3)
C2—C1—C6—C5	2.6 (4)	C9—C8—C15—N2	62.9 (3)
O1—C1—C6—C7	2.8 (4)	N1—C8—C15—C16	61.3 (3)
C2—C1—C6—C7	−176.8 (3)	C9—C8—C15—C16	−175.3 (2)
C8—N1—C7—C6	−179.3 (2)	C15—N2—C17—C18	−176.7 (2)
C5—C6—C7—N1	179.7 (3)	N2—C17—C18—C23	0.3 (5)
C1—C6—C7—N1	−0.9 (4)	N2—C17—C18—C19	179.0 (3)
C7—N1—C8—C9	117.0 (3)	C23—C18—C19—C20	1.7 (5)
C7—N1—C8—C15	−117.9 (3)	C17—C18—C19—C20	−177.1 (3)
N1—C8—C9—C10	−134.2 (3)	C18—C19—C20—C21	−1.8 (6)
C15—C8—C9—C10	101.9 (3)	C19—C20—C21—C22	1.3 (7)
N1—C8—C9—C14	46.5 (3)	C20—C21—C22—C23	−0.7 (6)
C15—C8—C9—C14	−77.4 (3)	C21—C22—C23—O2	179.4 (3)
C14—C9—C10—C11	0.0 (5)	C21—C22—C23—C18	0.5 (5)
C8—C9—C10—C11	−179.2 (3)	C19—C18—C23—O2	−179.9 (3)
C9—C10—C11—C12	0.0 (5)	C17—C18—C23—O2	−1.1 (5)
C10—C11—C12—C13	−0.1 (5)	C19—C18—C23—C22	−1.0 (5)
C11—C12—C13—C14	0.2 (5)	C17—C18—C23—C22	177.8 (3)

### 2-[(1E)-({1-(3-Chlorophenyl)-2-[(E)-(2-hydroxybenzylidene)amino]propyl}imino)methyl]phenol (I) Hydrogen-bond geometry (Å, º)

**Table:**

*D*—H···*A*	*D*—H	H···*A*	*D*···*A*	*D*—H···*A*
O1—H1···N1	0.82 (1)	1.88 (3)	2.596 (3)	146 (4)
O2—H2···N2	0.82 (1)	1.88 (3)	2.588 (3)	143 (4)
C16—H16*C*···O1^i^	0.96	2.52	3.448 (4)	161

Symmetry code: (i) *x*, *y*+1, *z*.

### 2-[(1E)-({1-(4-Chlorophenyl)-2-[(E)-(2-hydroxybenzylidene)amino]propyl}imino)methyl]phenol (II) Crystal data

**Table:**

C_23_H_21_ClN_2_O_2_	*F*(000) = 824
*M**_r_* = 392.87	*D*_x_ = 1.303 Mg m^−^^3^
Monoclinic, *P*2_1_/*c*	Mo *K*α radiation, λ = 0.71073 Å
*a* = 6.7923 (2) Å	Cell parameters from 9979 reflections
*b* = 20.8261 (8) Å	θ = 2.4–23.5°
*c* = 14.1744 (6) Å	µ = 0.21 mm^−^^1^
β = 92.435 (2)°	*T* = 296 K
*V* = 2003.26 (13) Å^3^	Block, brown
*Z* = 4	0.35 × 0.30 × 0.25 mm

### 2-[(1E)-({1-(4-Chlorophenyl)-2-[(E)-(2-hydroxybenzylidene)amino]propyl}imino)methyl]phenol (II) Data collection

**Table:**

Bruker Kappa APEXII CCD diffractometer	4182 independent reflections
Radiation source: fine-focus sealed tube	2599 reflections with *I* > 2σ(*I*)
Graphite monochromator	*R*_int_ = 0.044
ω and φ scan	θ_max_ = 26.6°, θ_min_ = 2.4°
Absorption correction: multi-scan (SADABS; Bruker, 2004)	*h* = −8→8
*T*_min_ = 0.927, *T*_max_ = 0.959	*k* = −26→26
42044 measured reflections	*l* = −17→17

### 2-[(1E)-({1-(4-Chlorophenyl)-2-[(E)-(2-hydroxybenzylidene)amino]propyl}imino)methyl]phenol (II) Refinement

**Table:**

Refinement on *F*^2^	2 restraints
Least-squares matrix: full	Hydrogen site location: mixed
*R*[*F*^2^ > 2σ(*F*^2^)] = 0.047	H atoms treated by a mixture of independent and constrained refinement
*wR*(*F*^2^) = 0.140	*w* = 1/[σ^2^(*F*_o_^2^) + (0.0484*P*)^2^ + 0.8955*P*] where *P* = (*F*_o_^2^ + 2*F*_c_^2^)/3
*S* = 1.08	(Δ/σ)_max_ < 0.001
4182 reflections	Δρ_max_ = 0.30 e Å^−^^3^
262 parameters	Δρ_min_ = −0.29 e Å^−^^3^

### 2-[(1E)-({1-(4-Chlorophenyl)-2-[(E)-(2-hydroxybenzylidene)amino]propyl}imino)methyl]phenol (II) Special details

**Table:**

Geometry. All esds (except the esd in the dihedral angle between two l.s. planes) are estimated using the full covariance matrix. The cell esds are taken into account individually in the estimation of esds in distances, angles and torsion angles; correlations between esds in cell parameters are only used when they are defined by crystal symmetry. An approximate (isotropic) treatment of cell esds is used for estimating esds involving l.s. planes.

### 2-[(1E)-({1-(4-Chlorophenyl)-2-[(E)-(2-hydroxybenzylidene)amino]propyl}imino)methyl]phenol (II) Fractional atomic coordinates and isotropic or equivalent isotropic displacement parameters (Å^2^)

**Table:**

	*x*	*y*	*z*	*U*_iso_*/*U*_eq_	
Cl1	0.78279 (15)	0.12547 (4)	0.58629 (6)	0.1006 (3)	
O1	0.2304 (3)	−0.08496 (8)	0.15527 (14)	0.0637 (5)	
H1	0.334 (3)	−0.0660 (13)	0.171 (2)	0.087 (10)*	
O2	0.2661 (3)	0.08746 (11)	0.18436 (16)	0.0770 (6)	
H2	0.370 (3)	0.0672 (14)	0.182 (2)	0.098 (12)*	
N1	0.5917 (2)	−0.06874 (9)	0.21615 (13)	0.0467 (5)	
N2	0.6144 (2)	0.05789 (9)	0.13809 (13)	0.0462 (4)	
C1	0.2600 (3)	−0.14842 (11)	0.16815 (16)	0.0482 (5)	
C2	0.1094 (4)	−0.19034 (13)	0.14300 (19)	0.0622 (7)	
H2A	−0.0098	−0.1747	0.1178	0.075*	
C3	0.1357 (5)	−0.25512 (14)	0.1552 (2)	0.0707 (8)	
H3	0.0332	−0.2831	0.1391	0.085*	
C4	0.3108 (5)	−0.27897 (13)	0.1909 (2)	0.0700 (8)	
H4	0.3275	−0.3230	0.1986	0.084*	
C5	0.4615 (4)	−0.23786 (12)	0.21510 (18)	0.0614 (7)	
H5	0.5805	−0.2543	0.2393	0.074*	
C6	0.4400 (3)	−0.17188 (10)	0.20426 (15)	0.0464 (5)	
C7	0.6050 (3)	−0.12931 (11)	0.22487 (15)	0.0478 (5)	
H7	0.7252	−0.1470	0.2452	0.057*	
C8	0.7683 (3)	−0.02938 (10)	0.22775 (15)	0.0452 (5)	
H8	0.8834	−0.0578	0.2314	0.054*	
C9	0.7673 (3)	0.01045 (11)	0.31730 (15)	0.0463 (5)	
C10	0.9417 (4)	0.03504 (14)	0.35463 (18)	0.0633 (7)	
H10	1.0577	0.0274	0.3239	0.076*	
C11	0.9481 (4)	0.07086 (14)	0.4368 (2)	0.0696 (7)	
H11	1.0674	0.0866	0.4617	0.084*	
C12	0.7775 (4)	0.08287 (12)	0.48094 (18)	0.0632 (7)	
C13	0.6031 (4)	0.06095 (13)	0.44453 (19)	0.0651 (7)	
H13	0.4870	0.0704	0.4742	0.078*	
C14	0.5981 (4)	0.02444 (13)	0.36300 (18)	0.0595 (6)	
H14	0.4779	0.0090	0.3386	0.071*	
C15	0.7810 (3)	0.01313 (11)	0.13977 (15)	0.0454 (5)	
H15	0.9041	0.0377	0.1442	0.054*	
C16	0.7758 (4)	−0.02621 (12)	0.04999 (17)	0.0566 (6)	
H16A	0.8808	−0.0571	0.0531	0.085*	
H16B	0.7916	0.0016	−0.0031	0.085*	
H16C	0.6517	−0.0481	0.0430	0.085*	
C17	0.6244 (3)	0.11048 (11)	0.09408 (15)	0.0459 (5)	
H17	0.7395	0.1207	0.0642	0.055*	
C18	0.4608 (3)	0.15533 (11)	0.08902 (16)	0.0481 (5)	
C19	0.4742 (4)	0.21199 (13)	0.03970 (19)	0.0697 (7)	
H19	0.5890	0.2213	0.0090	0.084*	
C20	0.3212 (6)	0.25477 (16)	0.0351 (3)	0.0959 (11)	
H20	0.3309	0.2927	0.0011	0.115*	
C21	0.1538 (6)	0.2408 (2)	0.0815 (3)	0.1000 (13)	
H21	0.0501	0.2700	0.0789	0.120*	
C22	0.1349 (4)	0.18543 (17)	0.1312 (2)	0.0796 (9)	
H22	0.0197	0.1771	0.1622	0.096*	
C23	0.2875 (3)	0.14176 (13)	0.13520 (18)	0.0582 (6)	

### 2-[(1E)-({1-(4-Chlorophenyl)-2-[(E)-(2-hydroxybenzylidene)amino]propyl}imino)methyl]phenol (II) Atomic displacement parameters (Å^2^)

**Table:**

	*U*^11^	*U*^22^	*U*^33^	*U*^12^	*U*^13^	*U*^23^
Cl1	0.1458 (8)	0.0881 (6)	0.0693 (5)	−0.0325 (5)	0.0213 (5)	−0.0277 (4)
O1	0.0522 (10)	0.0485 (11)	0.0897 (14)	0.0042 (9)	−0.0063 (9)	0.0005 (9)
O2	0.0553 (12)	0.0837 (15)	0.0935 (15)	−0.0041 (11)	0.0232 (10)	0.0027 (12)
N1	0.0449 (10)	0.0437 (11)	0.0516 (11)	−0.0004 (8)	0.0032 (8)	−0.0005 (9)
N2	0.0425 (10)	0.0489 (11)	0.0473 (11)	−0.0010 (8)	0.0039 (8)	0.0023 (9)
C1	0.0546 (13)	0.0437 (13)	0.0466 (13)	0.0012 (11)	0.0074 (10)	−0.0004 (10)
C2	0.0591 (15)	0.0630 (17)	0.0646 (16)	−0.0075 (13)	0.0039 (12)	−0.0038 (13)
C3	0.085 (2)	0.0631 (18)	0.0637 (17)	−0.0232 (15)	0.0040 (15)	−0.0013 (14)
C4	0.103 (2)	0.0419 (14)	0.0641 (17)	−0.0084 (15)	−0.0036 (15)	0.0040 (12)
C5	0.0808 (18)	0.0473 (15)	0.0553 (15)	0.0065 (13)	−0.0069 (13)	0.0069 (12)
C6	0.0596 (14)	0.0414 (13)	0.0384 (12)	0.0007 (10)	0.0032 (10)	0.0018 (9)
C7	0.0522 (13)	0.0484 (14)	0.0424 (12)	0.0061 (11)	−0.0011 (10)	0.0022 (10)
C8	0.0376 (11)	0.0473 (13)	0.0507 (13)	0.0025 (9)	0.0020 (9)	0.0011 (10)
C9	0.0475 (12)	0.0484 (13)	0.0430 (12)	−0.0037 (10)	0.0009 (10)	0.0056 (10)
C10	0.0507 (14)	0.0825 (19)	0.0568 (16)	−0.0062 (13)	0.0037 (11)	−0.0040 (14)
C11	0.0683 (17)	0.079 (2)	0.0609 (17)	−0.0178 (15)	−0.0056 (13)	−0.0072 (15)
C12	0.090 (2)	0.0511 (15)	0.0490 (15)	−0.0159 (14)	0.0090 (14)	0.0003 (12)
C13	0.0709 (17)	0.0623 (17)	0.0638 (17)	−0.0060 (13)	0.0219 (14)	−0.0050 (13)
C14	0.0521 (14)	0.0703 (17)	0.0568 (15)	−0.0080 (12)	0.0093 (11)	−0.0071 (13)
C15	0.0375 (11)	0.0535 (13)	0.0454 (13)	−0.0009 (10)	0.0049 (9)	0.0014 (10)
C16	0.0570 (14)	0.0645 (16)	0.0486 (14)	0.0080 (12)	0.0064 (11)	−0.0025 (12)
C17	0.0461 (12)	0.0503 (14)	0.0415 (12)	−0.0061 (10)	0.0048 (9)	−0.0024 (10)
C18	0.0539 (13)	0.0473 (13)	0.0427 (13)	0.0021 (11)	−0.0044 (10)	−0.0062 (10)
C19	0.088 (2)	0.0596 (17)	0.0606 (17)	0.0096 (15)	−0.0041 (14)	0.0024 (14)
C20	0.125 (3)	0.064 (2)	0.096 (3)	0.025 (2)	−0.025 (2)	0.0008 (18)
C21	0.092 (3)	0.092 (3)	0.112 (3)	0.046 (2)	−0.037 (2)	−0.037 (2)
C22	0.0538 (16)	0.090 (2)	0.094 (2)	0.0128 (16)	−0.0105 (14)	−0.035 (2)
C23	0.0504 (14)	0.0625 (16)	0.0610 (16)	0.0018 (12)	−0.0041 (11)	−0.0171 (13)

### 2-[(1E)-({1-(4-Chlorophenyl)-2-[(E)-(2-hydroxybenzylidene)amino]propyl}imino)methyl]phenol (II) Geometric parameters (Å, º)

**Table:**

Cl1—C12	1.736 (3)	C10—C11	1.383 (4)
O1—C1	1.348 (3)	C10—H10	0.9300
O1—H1	0.833 (10)	C11—C12	1.363 (4)
O2—C23	1.339 (3)	C11—H11	0.9300
O2—H2	0.822 (10)	C12—C13	1.351 (4)
N1—C7	1.270 (3)	C13—C14	1.383 (4)
N1—C8	1.456 (3)	C13—H13	0.9300
N2—C17	1.264 (3)	C14—H14	0.9300
N2—C15	1.465 (3)	C15—C16	1.513 (3)
C1—C2	1.380 (3)	C15—H15	0.9800
C1—C6	1.394 (3)	C16—H16A	0.9600
C2—C3	1.371 (4)	C16—H16B	0.9600
C2—H2A	0.9300	C16—H16C	0.9600
C3—C4	1.366 (4)	C17—C18	1.451 (3)
C3—H3	0.9300	C17—H17	0.9300
C4—C5	1.367 (4)	C18—C19	1.376 (3)
C4—H4	0.9300	C18—C23	1.400 (3)
C5—C6	1.389 (3)	C19—C20	1.368 (4)
C5—H5	0.9300	C19—H19	0.9300
C6—C7	1.449 (3)	C20—C21	1.368 (5)
C7—H7	0.9300	C20—H20	0.9300
C8—C9	1.516 (3)	C21—C22	1.361 (5)
C8—C15	1.535 (3)	C21—H21	0.9300
C8—H8	0.9800	C22—C23	1.378 (4)
C9—C14	1.374 (3)	C22—H22	0.9300
C9—C10	1.375 (3)		
			
C1—O1—H1	108 (2)	C13—C12—Cl1	119.1 (2)
C23—O2—H2	108 (2)	C11—C12—Cl1	120.1 (2)
C7—N1—C8	119.55 (18)	C12—C13—C14	119.7 (2)
C17—N2—C15	120.13 (18)	C12—C13—H13	120.2
O1—C1—C2	118.7 (2)	C14—C13—H13	120.2
O1—C1—C6	121.1 (2)	C9—C14—C13	121.3 (2)
C2—C1—C6	120.1 (2)	C9—C14—H14	119.4
C3—C2—C1	119.9 (3)	C13—C14—H14	119.4
C3—C2—H2A	120.1	N2—C15—C16	109.91 (18)
C1—C2—H2A	120.1	N2—C15—C8	108.07 (17)
C4—C3—C2	120.8 (3)	C16—C15—C8	111.79 (19)
C4—C3—H3	119.6	N2—C15—H15	109.0
C2—C3—H3	119.6	C16—C15—H15	109.0
C3—C4—C5	119.7 (3)	C8—C15—H15	109.0
C3—C4—H4	120.1	C15—C16—H16A	109.5
C5—C4—H4	120.1	C15—C16—H16B	109.5
C4—C5—C6	121.2 (2)	H16A—C16—H16B	109.5
C4—C5—H5	119.4	C15—C16—H16C	109.5
C6—C5—H5	119.4	H16A—C16—H16C	109.5
C5—C6—C1	118.3 (2)	H16B—C16—H16C	109.5
C5—C6—C7	120.4 (2)	N2—C17—C18	121.7 (2)
C1—C6—C7	121.2 (2)	N2—C17—H17	119.2
N1—C7—C6	122.5 (2)	C18—C17—H17	119.2
N1—C7—H7	118.8	C19—C18—C23	119.0 (2)
C6—C7—H7	118.8	C19—C18—C17	120.6 (2)
N1—C8—C9	111.71 (17)	C23—C18—C17	120.3 (2)
N1—C8—C15	107.90 (17)	C20—C19—C18	121.1 (3)
C9—C8—C15	111.53 (18)	C20—C19—H19	119.5
N1—C8—H8	108.5	C18—C19—H19	119.5
C9—C8—H8	108.5	C21—C20—C19	118.9 (3)
C15—C8—H8	108.5	C21—C20—H20	120.5
C14—C9—C10	117.6 (2)	C19—C20—H20	120.5
C14—C9—C8	122.9 (2)	C22—C21—C20	121.8 (3)
C10—C9—C8	119.5 (2)	C22—C21—H21	119.1
C9—C10—C11	121.4 (2)	C20—C21—H21	119.1
C9—C10—H10	119.3	C21—C22—C23	119.6 (3)
C11—C10—H10	119.3	C21—C22—H22	120.2
C12—C11—C10	119.3 (2)	C23—C22—H22	120.2
C12—C11—H11	120.4	O2—C23—C22	118.7 (3)
C10—C11—H11	120.4	O2—C23—C18	121.7 (2)
C13—C12—C11	120.8 (2)	C22—C23—C18	119.6 (3)
			
O1—C1—C2—C3	179.9 (2)	C11—C12—C13—C14	1.8 (4)
C6—C1—C2—C3	1.2 (4)	Cl1—C12—C13—C14	−177.0 (2)
C1—C2—C3—C4	−1.0 (4)	C10—C9—C14—C13	−1.1 (4)
C2—C3—C4—C5	0.3 (4)	C8—C9—C14—C13	−180.0 (2)
C3—C4—C5—C6	0.1 (4)	C12—C13—C14—C9	−0.7 (4)
C4—C5—C6—C1	0.2 (4)	C17—N2—C15—C16	−81.7 (2)
C4—C5—C6—C7	−176.2 (2)	C17—N2—C15—C8	156.0 (2)
O1—C1—C6—C5	−179.5 (2)	N1—C8—C15—N2	65.8 (2)
C2—C1—C6—C5	−0.8 (3)	C9—C8—C15—N2	−57.2 (2)
O1—C1—C6—C7	−3.1 (3)	N1—C8—C15—C16	−55.3 (2)
C2—C1—C6—C7	175.6 (2)	C9—C8—C15—C16	−178.30 (17)
C8—N1—C7—C6	−173.12 (19)	C15—N2—C17—C18	178.55 (19)
C5—C6—C7—N1	−179.8 (2)	N2—C17—C18—C19	−179.2 (2)
C1—C6—C7—N1	3.9 (3)	N2—C17—C18—C23	1.5 (3)
C7—N1—C8—C9	−110.4 (2)	C23—C18—C19—C20	−0.2 (4)
C7—N1—C8—C15	126.6 (2)	C17—C18—C19—C20	−179.5 (3)
N1—C8—C9—C14	−20.7 (3)	C18—C19—C20—C21	0.7 (5)
C15—C8—C9—C14	100.2 (2)	C19—C20—C21—C22	−0.6 (5)
N1—C8—C9—C10	160.5 (2)	C20—C21—C22—C23	−0.2 (5)
C15—C8—C9—C10	−78.6 (3)	C21—C22—C23—O2	−179.9 (3)
C14—C9—C10—C11	2.0 (4)	C21—C22—C23—C18	0.7 (4)
C8—C9—C10—C11	−179.1 (2)	C19—C18—C23—O2	−179.9 (2)
C9—C10—C11—C12	−1.0 (4)	C17—C18—C23—O2	−0.6 (3)
C10—C11—C12—C13	−0.9 (4)	C19—C18—C23—C22	−0.6 (4)
C10—C11—C12—Cl1	177.9 (2)	C17—C18—C23—C22	178.8 (2)

### 2-[(1E)-({1-(4-Chlorophenyl)-2-[(E)-(2-hydroxybenzylidene)amino]propyl}imino)methyl]phenol (II) Hydrogen-bond geometry (Å, º)

Cg is the centroid of the C18–C23 ring.

**Table:**

*D*—H···*A*	*D*—H	H···*A*	*D*···*A*	*D*—H···*A*
O1—H1···N1	0.83 (1)	1.83 (2)	2.589 (2)	150 (3)
O2—H2···N2	0.82 (1)	1.81 (2)	2.557 (3)	150 (3)
C14—H14···N1	0.93	2.52	2.845 (3)	101
C5—H5···*Cg*^i^	0.93	2.76	3.449 (3)	132

Symmetry code: (i) −*x*+1, *y*−1/2, −*z*+1/2.
